# An Exploratory Study Examining Obesity Risk in Non-Obese Mothers of Young Children Using a Socioecological Approach

**DOI:** 10.3390/nu10060781

**Published:** 2018-06-17

**Authors:** Jennifer Martin-Biggers, Virginia Quick, Kim Spaccarotella, Carol Byrd-Bredbenner

**Affiliations:** 1 Department of Nutritional Sciences, Rutgers University, 26 Nichol Avenue, New Brunswick, NJ 08901, USA; jmartinbiggers@rutgers.edu (J.M.-B.); bredbenner@sebs.rutgers.edu (C.B.-B.); 2 Department of Biological Sciences, Kean University, 1000 Morris Avenue, Union, NJ 07082, USA; kspaccar@kean.edu

**Keywords:** obesity risk, mothers, women, young children, socioecological

## Abstract

This cross-sectional, exploratory study aimed to (1) develop an obesity risk score using a comprehensive set of variables assessing mothers’ intrapersonal weight-related characteristics and those of their homes’ interpersonal and physical environments, and (2) determine how weight-related characteristics differ by obesity risk level. U.S. mothers (*N* = 550) of preschool-aged children completed an online survey that assessed maternal self-report weight status, sociodemographics, health-related characteristics, and maternal intrapersonal and their homes’ interpersonal and physical environment weight-related characteristics. Binomial logistic regression analysis identified variables significantly associated with obesity. Scores for all obesity risk variables were summed to create a weighted obesity risk score for non-obese participants (*n* = 386). Analysis of variance and Tukey post-hoc tests determined how non-obese mothers’ sociodemographic, health-related, and intrapersonal and their homes’ interpersonal and physical environment characteristics differed among obesity risk score tertiles. Results revealed that eight variables explained 53 percent of maternal obesity risk, including African American race, lower education level, more children in household, poorer maternal health, higher weight teasing history, higher body dissatisfaction, primary relative with obesity, and greater concern about children’s overweight risk. Non-obese mothers in the highest obesity risk tertile had greater food insecurity risk, lower family affluence, worse sleep quality, less fruit/vegetable availability, and reported less frequent modeling of healthy behaviors and more family conflict. In conclusion, eight characteristics that explained more than half of the risk for obesity in non-obese mothers of young children, may help healthcare professionals identify mothers at increased risk of obesity and offer preventive care early.

## 1. Introduction

Recent data from the National Health and Nutrition Examination Survey (NHANES) indicate that nearly 34.9 percent of U.S. adults are obese [1]. It is no longer debated that obesity and its comorbidities are significantly impacting Americans both in financial and quality of life costs. A systematic review of 33 studies found that the overall estimated medical costs of obesity accounted for approximately 10 percent of all medical spending in the United States [2]. The physical health consequences of obesity are numerous and include effects on the pulmonary, orthopedic, neurological, gastroenterological, endocrine, and cardiovascular systems, as well as causing systemic inflammation, thereby greatly impacting quality of life [3,4,5,6].

The increase in obesity rates in the U.S. likely reflects changes in environmental factors and lifestyle choices related to increased energy intake and inadequate energy expenditure, rather than genetic changes because of the slow rate at which population-wide genetic changes occur [7,8,9]. Changes in lifestyles and the environment that have occurred in tandem with the increase in obesity include ready availability of food and shifting dietary patterns, which have led to an increase in calorie intake [10,11], combined with a decline in energy expenditure associated with a sedentary lifestyle [12]. Macro-level factors have a more indirect (yet important) role in influencing behaviors and include social norms, agriculture policies, economic policies, advertising, and more. Factors that are more directly influenced by an individual include his or her physical and social environments and personal factors (e.g., home environment, skills, behaviors).

In recent years, health behavior change experts have recognized the influence of factors in the physical environment, as well as in the social environment, on obesity and health outcomes [13]. This ecological approach to public health issues posits that an individual’s motivation and skills alone are not adequate to facilitate behavior change; environments also need to support and facilitate the practice of healthful behaviors [14,15,16]. Reciprocal determinism, a construct of the Social Cognitive Theory, describes how a person’s characteristics and behaviors, as well as the physical and social environment where behaviors occur, mutually affect each other [17]. Environments not supportive of weight-management behaviors make it difficult to engage in practices that prevent unhealthy weight gain. Research has increasingly provided evidence that environmental factors significantly influence diet, physical activity, and obesity in adults [15,18,19] and children [16,20,21,22], yet their relative contribution to obesity risk remains unknown.

The home environment deserves in-depth study given its potential influence on health behaviors [23]. An understanding of factors in the home environment associated with obesity could assist healthcare providers, researchers, parents, and caregivers in creating home environments that support healthy weights for the whole family. Mothers tend to be food gatekeepers in the home and, thus, are well suited to providing an appraisal of their homes’ social (interpersonal) and physical environment characteristics [23,24]. Mothers also are children’s role models and often the household food decision-maker, thus mothers’ own intrapersonal cognitions, behaviors, and weight status play a role in weight-related decisions affecting the entire family [23,24]. Moreover, behaviors directly affecting weight are developed during childhood and track into adulthood [25,26,27,28], thereby making it vital to identify factors affecting maternal obesity risk and address them in obesity prevention interventions. A deeper understanding of the full interplay of intrapersonal and interpersonal characteristics and behaviors along with environments could provide a more complete picture of the aspects of the home environment that may place mothers of young children at risk for weight gain. A method for assessing risk for weight gain could enable health care providers and researchers to tailor and design more effective nutrition education.

Health experts acknowledge that lifestyles and the environment play a role in obesity risk [29]; however, few studies have considered these factors when examining obesity risk and none could be located that examined a comprehensive array of factors. Thus, the aims of this exploratory study were to (1) develop an obesity risk score using a comprehensive set of variables assessing mothers’ own intrapersonal weight-related characteristics as well as the weight-related characteristics of their homes’ interpersonal and physical environments, and (2) determine how these weight-related characteristics differ among levels of obesity risk.

## 2. Materials and Methods

This research was approved by the Institutional Review Board at Rutgers University. All participants gave informed consent.

### 2.1. Sample

Survey Sampling International (SSI), a global research company whose services include survey sample participant recruitment (www.surveysampling.com), was utilized. SSI panel members received invitations to participate in an online survey that would help researchers create “a program to help parents to build healthier kids”. The goal was to recruit mothers of children in the target age range who had most of the food decision-making authority in their households to adequately capture the most representative responses. To meet eligibility requirements, panel members had to be female, between 18- and 45-years-old, have one or more children 2- to 5-years-old, and be the household’s primary food gatekeeper (i.e., made most or all food purchasing and preparation decisions). As an incentive to complete the cross-sectional online survey, participating mothers accrued points from SSI that were redeemable for gifts. A total sample size of 384 was estimated based on a 95% confidence interval, and total population of women in the U.S. that are 20–45 years old using 2010 U.S. Census data [30].

### 2.2. Instrument

Development and content of the online Home Obesogenicity Measure of EnvironmentS (HOMES) survey is described in detail elsewhere [31,32,33]. In summary, development began with a comprehensive literature review to identify salient weight-related demographic, environmental, behavioral, and psychographic characteristics. Self-report scales assessing these characteristics, preferably those previously used and validated with a diverse sample of U.S. adults, also were identified. When multiple scales for assessing a characteristic were found, each was reviewed to determine which was most relevant to the study sample, easy to administer and score, and had good reliability and validity. If an instrument assessing a characteristic of interest or fitting the needs of the study could not be located in the literature, items were developed *de novo*. For scales with items substantially modified from their original form or developed *de novo*, standard processes for developing and refining scales were applied [34]. That is, experts (*n* = 5) in subject matter areas appropriate to the scale content (e.g., nutrition, physical activity, psychology, child development, obesogenic environment), psychometrics, and survey design iteratively reviewed and refined them to ensure scale clarity and content validity [34,35]. The items then underwent cognitive testing with participants who were similar to the study population to assess whether the items were interpreted as intended [35] and to determine ways to reduce participant burden and increase understandability and acceptability [36].

After refinement, the scales were consolidated into a single survey that was posted online (using Qualtrics^®^). The survey was pilot-tested with 48 participants whose characteristics were the same as those of the target audience for the final study (but were not participants in the final sample) to gauge completion time, identify further refinements needed to improve clarity and ease of completion, ensure protocols for scoring of scales were accurate, and conduct preliminary psychometric analyses. The survey was administered online for ease of data collection and convenience to participants, to help reduce the potential for social desirability bias that can occur during in-person administration, and to increase researcher ability to reach individuals who would be otherwise difficult to access (i.e., distance from researchers or limited time to meet in-person) [37,38].

The HOMES survey included an array of measures that focused on mother’s intrapersonal weight-related characteristics and their homes’ interpersonal and physical environment characteristics and yielded 79 variables. Table 1 lists the variables in the HOMES survey considered in the creation of the obesity risk score, including number of items, possible score range, and Cronbach’s alpha (when applicable).

### 2.3. Sociodemographics and Health-Related Characteristics

Variables addressing maternal sociodemographic data included race/ethnicity, education level, number of children in the household, family affluence [39,40], maternal employment, and food insecurity risk [41]. There were eight variables examining health-related characteristics (e.g., general health status [42,43], health-related quality of life [42,43], depression severity [44], age at birth of first child, perception of weight teasing history [45], body dissatisfaction [46], primary relative with obesity and/or obesity. 

#### 2.3.1. Weight Status

Self-reported heights and weights of participants were used to calculate body mass index (BMI) (weight (kg)/(height (m^2^)). Participants also reported their children’s height, weight, sex, and age which were used to calculated their children’s BMI percentile [91].

#### 2.3.2. Intrapersonal Characteristics

Mothers’ personal weight-related behaviors (e.g., physical activity level and screentime [47,48,49], sleep quality and duration [50,51], fruit/vegetable intake [52,53,54], percent calories from fat [52,53,54], milk intake [55,56], sugar-sweetened beverage intake [55,56]) accounted for eight variables. Scales evaluating maternal eating styles (e.g., disinhibited eating [57,58], emotional eating [57,58], dietary restraint eating [57,58], adventurous eating [59,60,61]) generated four variables. Five variables were produced from measures of mothers’ self-perceptions (i.e., personal organization [62], need for cognition [63,64], parenting self-efficacy [65,66], stress management [67], stress management self-efficacy). Cognitions related to children’s weight (i.e., belief that chubby kids are healthy, concern about own children’s overweight risk [68]) were assessed with two scales. The value of engaging in healthy behaviors for self and child (i.e., importance placed on physical activity, encouragement and facilitation of children’s physical activity, importance of modeling physical activity to children, frequency of engaging in active play with children, parent modeling of healthy eating, parenting cognitions and behaviors associated with children’s television viewing) [31,69,70,71,72] was examined with 12 measures, each generating a variable.

#### 2.3.3. Home Interpersonal Characteristics

Assessments of mothers’ child feeding behaviors (e.g., food restriction, pressuring, rewarding) [68,69,70,71,73,74,75] yielded 10 variables. Family meal patterns (e.g., frequency and location of meals) [70,76,77,78,79,80] resulted in nine variables. Scales assessing family functioning and engagement (e.g., family support for healthy behaviors [81,82,83], family conflict and lack of cohesion [84,85,86], household organization [87,88]) included five variables. 

#### 2.3.4. Home Physical Environment

Appraisal of the home physical environment’s accessibility to and availability of physical activity and sedentary activity opportunities (e.g., physical activity supports, media devices in the home, children’s TV accessibility) [69,70,71,72,82,89] contributed five variables. Measures of household food availability (e.g., fruit/vegetables, sugar-sweetened beverages) [52,55,56,90] generated 4 variables.

### 2.4. Data Analysis

Internal consistencies of all measures, when applicable, were calculated using Cronbach’s alpha. Descriptive statistics of all variables in the total sample were evaluated in four steps before further development of the obesity risk score. First, Spearman rank order correlations of all variables, except mothers’ and children’s weight status, were examined for multicollinearity. In the correlation matrix, race/ethnicity was categorized into two dichotomous variables (i.e., white or non-white, black or non-black) and education level was dichotomized into low (some college or less) or high (baccalaureate degree or higher). Variables that were intercorrelated (i.e., *r* > 0.50) were reviewed to select a single variable from among them to use in further analyses. The criterion for selecting a single variable from among those that were intercorrelated was that the variable under consideration had to be significantly correlated (*p* < 0.05) with maternal BMI. If none of the intercorrelated variables met this criterion, none were considered in further analyses. If more than one variable met this criterion, the variable with the highest correlation (absolute value) with maternal BMI was selected. In the second step of data analysis, binomial logistic regression was conducted to identify variables significantly associated with obesity. Variables remaining after the first step of the data analysis served as independent variables. The dependent variable was maternal weight status of obese (i.e., BMI ≥ 30) vs. non-obese (i.e., BMI ≤ 30). In step three of data analysis, the significant obesity risk variables identified in step two were again entered into the binomial logistic regression analysis to determine the best model fit and confirm results. In step four, data for non-obese mothers’ were extracted from the data set and median scores were calculated for all variables found to be significantly associated with obesity. 

To create the weighted obesity risk score, each non-obese mother was assigned a score for each obesity risk variable found in the regression model, as stated above, to be significantly associated with obesity. If a participant’s score for an obesity risk variable was above the median for continuous variables or mothers had the characteristic for dichotomous variables, the score assigned for the variable was the beta coefficient value generated by the binomial logistic regression (reflecting an increased risk for obesity); if a particiapant’s score was below the median for continuous variables or mothers did not have the characteristic for dichotomous variables, thereby indicating reduced obesity risk, a score of 0 was awarded for that variable. Scores for all obesity risk variables were then summed to yield a participant’s total weighted obesity risk score. 

To determine how non-obese mothers differed by obesity risk level, they were assigned to groups based on their obesity risk score tertile (i.e., low, moderate, and high risk). ANOVA and Tukey post-hoc tests were conducted to determine how maternal sociodemographic, health-related, and intrapersonal and their homes’ interpersonal and physical environment characteristics differed among and between obesity risk score tertiles. For variables that were statically significant (*p* < 0.05), effect sizes were estimated by examining partial Eta squared, Analyses were performed with SPSS software version 24.0 (IBM corporation, Chicago, IL, USA). Given the number of variables investigated, significance level for main effects was set at 0.01 to reduce the risk of type 1 errors while maintaining sufficient power. Significance level for post-hoc procedures was set at *p* < 0.05. 

## 3. Results

Out of 910 participants who responded to the online survey, only 550 participants were eligible and completed the survey (i.e., 188 did not complete the survey, 96 did not meet inclusion criteria, and 76 had implausible responses (e.g., multiple items had the same answers)) with a survey response rate of 60%. Most participants (*n* = 550; mean age 32.25 ± 5.81 SD years) were white (72%) with some post-secondary education (82%). Nearly all measures had good to excellent internal consistency as determined by Cronbach’s alpha (see Table 1). Spearman rank order correlation coefficients of the study variables revealed that several variables were multicollinear. Table 2 lists each group of multicollinear variables and the variable selected from each group based on the criteria previously described in the Data Analysis section.

Of the original 79 variables examined, scores for the 62 variables that were not highly intercorrelated were retained for further analysis. Binomial logistic regression on the dichotomous dependent variable of non-obese (*n* = 386) vs obese (*n* = 164) weight status revealed that all of the variables combined explained 64 percent of maternal obesity risk, with 12 of the variables being significantly associated with obesity risk. The 12 significant obesity risk variables were retained and again subjected to binomial logistic regression resulting in a final model with 8 variables explaining 53 percent of maternal risk for obesity (Table 3). Three of the obesity risk variables were dichotomous variables (i.e., being black or African American, having lower education level, having a primary relative with a history of obesity) and five were continuous variables (i.e., larger number of children in household, poorer general health rating, higher perceived weight teasing history, greater body dissatisfaction, more concern about their children’s overweight risk). Using the variable scoring procedure described in the Data Analysis section, the weighted obesity risk score averaged 1.66 ± 0.98 SD and ranged from 0 to 5.49.

ANOVA and Tukey post-hoc tests comparing obesity risk scores by tertiles, as shown in Table 4, revealed significant differences (*p* < 0.001) among low-, moderate-, and high risk groups with a large effect size (i.e., η^2^ = 0.833) for the obesity risk score, as estimated by partial Eta squared. A review of the characteristics related to sociodemographic variables, weight status, and health showed that non-obese mothers in the low obesity risk tertile had significantly higher family affluence (*p* < 0.001), lower food security risk (*p* = 0.006), and higher BMIs (*p* < 0.001) than those in the high obesity risk tertile. Additionally, the high obesity risk tertile reported significantly more days of “not good” health in the past month (*p* < 0.001), younger age at birth of first child (*p* = 0.007), and higher depression severity (*p* < 0.001) compared with those in lower obesity risk tertiles. There were no significant child BMI percentile differences among obesity risk tertiles although there was an increasing trend.

An examination of maternal intrapersonal characteristics showed that those in the high obesity risk tertile were significantly more likely to report worse sleep quality (*p* < 0.001), greater emotional eating (*p* < 0.001), perceive themselves as having less personal organization (*p* < 0.001), lower confidence in parenting (*p* = 0.004), and poorer stress management skills (*p* < 0.001) than those in the lower obesity risk tertiles. Compared to the high obesity risk tertile, mothers in the low obesity risk tertile were significantly more likely to value the importance of physical activity for self (*p* < 0.001) and child (*p* = 0.006), value the importance of modeling physical activity (*p* = 0.01), frequently model physical activity (*p* < 0.001) and less frequently model sedentary activity (*p* = 0.007), model healthy eating (*p* = 0.003), and place more child limits on children’s TV program choices (*p* = 0.01). None of the other intrapersonal characteristics differed significantly among obesity risk tertile groups.

The only interpersonal characteristics that differed significantly among obesity risk tertile groups were the frequency of family meals eaten at the kitchen or dining table and family conflict and lack of cohesion. That is, mothers in the high obesity risk tertile reported eating significantly (*p* = 0.006) fewer family meals at a kitchen or dining table and had more family conflict and less cohesion (*p* = 0.005) compared with those in the low obesity risk tertile. 

Household fruit and vegetable availability was the only home physical environment characteristic that differed significantly among obesity risk tertiles. That is, mothers in the low obesity risk tertile had greater household availability of fruits and vegetables (*p* = 0.013) than those in the high obesity risk tertile. Estimated effect sizes, as determined by partial Eta squared, for the significantly different intrapersonal, interpersonal, and home environment characteristics were low.

## 4. Discussion

This study developed an obesity risk score for non-obese mothers of young children using a comprehensive array of sociodemographic and weight-related intrapersonal, interpersonal, and home environmental characteristics. The eight characteristics comprising the obesity risk score may help health professionals identify non-obese mothers with young children at increased risk for obesity and provide early obesity prevention intervention. 

The eight independent variables identified in this study explained over half of maternal risk for obesity have been shown in other studies to be strongly associated with obesity. That is, women of African American race are more likely to be overweight or obese than other racial and ethnic groups [1]; lower education attainment is associated with overweight and obesity [1,83]; obese adults have more chronic disease [92,93] and report poorer health [94]; obese women are more likely to report being teased growing up [95,96]; and body shape dissatisfaction is associated with overweight and obesity [97,98]. The study reported here also provides insight into variables that are associated with obesity which are not changeable through nutrition and health promotion programs (e.g., race, family history, affluence), and those which are, yet are rarely targeted in nutrition education interventions (e.g., family conflict, body dissatisfaction). 

Examining non-obese mothers by high, moderate, and low obesity risk tertiles provided an efficient means for exploring how risk level was associated with interpersonal, intrapersonal, and environmental characteristics. Several sociodemographic variables, health-related assessments, and intrapersonal, and interpersonal characteristics were associated with high obesity risk score among non-obese mothers whereas home physical environment factors tended to not be associated with obesity risk. Trends in sociodemographic and health-related characteristics noted among participants at high risk for obesity (e.g., food insecurity, lower family affluence, more unhealthy days in past month, and greater depression severity) mirror national data [1,83]. For instance, mothers in the high obesity risk group reported less family affluence and greater depression severity, which is consistent with literature indicating obesity rates are higher among adult women with low socio-economic status [99] and obese women are more likely to suffer from depression (although this association may be bi-directional) [100,101].

High obesity risk mothers did not significantly differ from lower obesity risk moms in their physical activity and screentime behaviors possibly because at risk, but not obese, women are not yet hindered by their weight interfering with their activity level. These findings suggest that activity level may not be a significant risk factor for maternal obesity but is associated with obesity weight status, raising the question of reverse causality. That is, are obese mothers inactive and more sedentary [102] because their weight inhibits physical activity instead of inactivity causing weight gain; or, are there other factors contributing to these relationships, such as the environmental supports for physical activity? Environments that promote access to and availability of physical activity are associated with more physical activity behaviors [103,104,105,106,107]. However, a recent systematic review has found inconsistent results in the associations between the built physical environment and obesity in adults [108], perhaps due to the great variation in metrics used and differing contexts of prior studies [108]. 

There is growing interest in the associations of sleep duration and quality with weight [109]. In the present study, poor sleep quality was significantly more prevalent in high obesity risk mothers. Although non-significant, the number of total hours slept each night linearly decreased as obesity risk increased, with those in the high obesity risk group receiving less than the recommended total sleep hours per night for adults of 7 to 9 h [110]. These findings suggest the need for continued investigation of the mechanisms of how sleep may be related to weight gain, and indicate the importance of including sleep management in weight-management interventions. 

A linear trend showed that high obesity risk mothers were less active with their children and allowed children to have more minutes of screentime daily than lower obesity risk mothers. This finding is similar to reports of associations between maternal characteristics, child activity, and sedentary behaviors [111,112], and further highlights the importance of maternal modeling, encouraging, and facilitating physical activity for their children [69]. Not surprisingly, low obesity risk mothers engaged in significantly more modeling of both physical activity and less sedentary behaviors. Additionally, mothers at low obesity risk were less likely to model unhealthy emotional eating behaviors for their children. Parents and caregivers act as powerful socialization agents and serve as models of eating that children learn to emulate [113]. Future work should explore how low obesity risk mothers’ modeling of healthy behaviors affects the health and weight status of their children. 

Home food availability, parental diet, and family eating habits are associated with diet quality of children [114,115]. In this study, high obesity risk moms had less household availability of fruits and vegetables and ate fewer family meals at a kitchen or dining room table which may have influenced their child’s diet quality and weight status. Although non-significant, there was a positive linear trend in child BMI percentile and maternal obesity risk score. Studies on weight-resilience (i.e., maintaining a healthy weight despite living in an obesogenic environment) suggest that homes with healthy weight children and teens are more likely to have healthier food options available and limit access to unhealthy foods [116,117]. In the study reported here, only a non-significant linear trend occurred, with mothers at low obesity risk limiting children’s access to low nutrient dense foods and storing nutrient dense foods in a manner that was clearly visible to children. Thus, future interventions should consider targeting family nutrition education that encourages positive changes in the home food supply and healthful dietary practices in the home.

This study found a non-significant decreasing trend in the relationships among obesity risk tertiles and family meal frequency. Other studies have found cross-sectional associations of family meal frequency were inversely associated with obesity in adolescents, but longitudinal analyses have not corroborated those results [118,119,120]. The results of this study contribute to the mixed associations among family meals and weight status [121]. It may be that characteristics of the family meal environment are confounding potential associations. For example, non-obese mothers at high risk for obesity reported significantly more family conflict and lack of cohesion. Having poor family functioning (i.e., more conflict and less cohesion) during mealtimes may lead to less frequent family meals, especially when there is a negative meal atmosphere [122,123,124]. Further research examining the influences of family dynamics with family meal frequency and weight-related behaviors are warranted [122,125].

The relationships between home food availability and maternal dietary intake are not clear. For instance, sugar-sweetened beverage intake was relatively lower among low obesity risk mothers yet household availability of sugar-sweetened beverages was similar in households across maternal obesity risk tertiles. Mothers at low obesity risk ate relatively more fruits and vegetables and had significantly greater household availability of fruits and vegetables than those of higher obesity risk. Whether foods were eaten from household food stores or outside the home was not investigated; the incongruence of beverage intake and household availability may indicate these beverages were consumed at home as well as outside the home, whereas the relative consistency between fruit and vegetable intake and household food supplies may indicate these foods are typically eaten at home. Further research is needed to understand where food and beverages are typically consumed and the impact of consumption location on household food supplies, overall family dietary intakes, and obesity risk.

A major strength of this study was the use of a socioecological framework to guide the comprehensive selection of constructs obesity risk in mothers of young children. A systematic method [34] was used to examine the potential scales for application to the study population (i.e., mothers and young children of varying races/ethnicities and educational attainment). Use of reliable and valid scales is vital to ensure the most accurate responses. In this survey, previously validated and refined tools were used when possible, and nearly all had good to high internal consistency with the study sample. Despite the strengths of this study, it is important to note its limitations. Inference of causality of observed associations cannot be made due to the cross-sectional study design. In addition, the study sample only included mothers of preschool-aged children in the U.S. with greater educational attainment than the national average, so findings may not be generalizable to mothers with lower education levels or mothers with children of different ages or those residing in other countries. This study also did not evaluate characteristics and behaviors of other family or friends or environments outside of the home environment. Although the estimated effect size for the obesity risk score was high, the significantly different intrapersonal, interpersonal, and home environment characteristics that occurred across obesity risk tertiles had low effect sizes. Lastly, data collected from participants were self-reported and may be subject to both reporting error and bias. However, heights and weights self-reported by adults are highly correlated with measured heights and weights [126] and online data collection offers privacy and relative anonymity that may improve veracity when answering questions [127,128,129].

## 5. Conclusions

In conclusion, this exploratory study identified eight characteristics that, together, explain more than half of the risk of obesity in non-obese mothers of young children. These eight characteristics may help healthcare professionals identify mothers at increased risk of obesity and offer preventive care early and more specifically tailor care (e.g., psychological assistance for those with body dissatisfaction). Many of the eight characteristics are not usually assessed in clinical practice, but are simple to assess and may yield valuable obesity risk information to healthcare providers. In addition, nutrition communication and health promotion professionals can apply the findings by targeting intervention efforts to those at increased risk and expanding intervention content to address topics not typically addressed in obesity prevention programs, such as strategies for managing family conflicts and changing the home food environment. Further research with larger, more diverse samples who are longitudinally assessed is needed to confirm the results of this study. Additional research to clarify the contribution of early identification of those at high risk for obesity and inclusion of new topics in obesity prevention programs also is warranted.

## Figures and Tables

**Table 1:** Description of Sociodemographic, Intrapersonal, Interpersonal and Home Environment Characteristics of Participants (*N* = 550).

Measure	# Items	Possible Score Range	Scale Type	Cronbach’s α	Mean ± SD or *N* (%)
Sociodemographic Characteristics					
Race/Ethnicity	1	n/a	categorical response	*	
White					396 (72.0)
Black or African American					52 (9.5)
Hispanic					25 (4.5)
Multi-racial					43 (7.8)
Asian					31 (5.6)
Other					3 (0.6)
Education Level	1	n/a	categorical response	*	
High School or less					99 (18.0)
Some college or technical/Associate’s degree					245 (44.5)
Bachelor’s degree or higher					206 (37.5)
Maternal Employment	1	n/a	categorical response	*	
Do not work					303 (55.1)
Part-time (less than 30 h/week)					103 (18.7)
Full-time (30 or more h/week)					143 (26.0)
Number of Children in Household	1	0 to more than 6	Total #	*	2.20 ± 1.01
Family Affluence Score [39,40]	3	0 to 7	varies per item ^A^	*	5.61 ± 1.56
Food Insecurity Risk [41]	2	1 to 4	4-point agreement rating ^B^	0.84	2.04 ± 1.91
Weight Status					
Mother’s BMI	1	n/a	Self-reported height/weight	*	27.69 ± 7.90
Child’s BMI percentile (*n* = 339)	1	n/a	Self-reported height/weight/sex/age	*	63.93 ± 34.93
Health-Related Assessments					
General Health Rating [42,43]	1	1 to 5	5-point excellence rating ^C^	*	3.52 ± 0.87
Health-related Quality of Life (# of unhealthy days) [42,43]	1	0 to 30	days/month	*	2.89 ± 4.56
Depression Severity [44]	2	1 to 4	4-point occurrence rating ^D^	0.81	1.05 ± 1.44
Age at Birth of First Child (years)	1	n/a	years	*	24.46 ± 5.39
Perception of Weight Teasing History [45]	3	1 to 5	5-point frequency rating ^E^	0.95	1.84 ± 1.15
Body Dissatisfaction [46]	1	1 to 4	4-point frequency rating ^F^	*	2.58 ± 1.10
Primary Relative with History of Obesity (% yes)	1	0 or 1	yes/no	*	207 (37.6)
Primary Relative with History of Diabetes (% yes)	1	0 or 1	yes/no	*	140 (25.5)
Intrapersonal Characteristics					
Maternal Weight-Related Behaviors					
Physical Activity Level [47,48,49]	3	0 to 42	8-point exercise scale ^G^	*	15.44 ± 9.98
Screentime	1	0 to 1440	minutes/day	*	273.52 ± 253.99
Sleep Duration	1	0 to 24	hours/day	*	7.11 ± 1.84
Sleep Quality [50,51]	1	1 to 5	5-point rating ^H^	*	3.24 ± 0.89
Fruit and Vegetable Intake [52,53,54]	10	0 to 12.17	6-point servings ^I^ eaten per day scale	*	4.56 ± 2.22
% Calories from Total Fat [52,53,54]	17	0 to 100	5-point servings eaten scale ^J^	*	37.4 ± 5.91
Milk [55,56]	1	0 to 8	9-point servings drank per day scale ^K^	*	3.95 ± 3.08
Sugar-Sweetened Beverage [55,56]	4	0 to 4.6	9-point servings drank per day scale ^K^	*	0.89 ± 0.88
Maternal Eating Styles					
Disinhibited Eating [57,58]	3	1 to 4	4-point agreement rating ^B^	0.81	1.96 ± 0.76
Emotional Eating [57,58]	3	1 to 4	4-point agreement rating ^B^	0.75	2.07 ± 0.88
Dietary Restraint Eating [57,58]	4	1 to 4	4-point agreement rating ^B^	0.74	2.42 ± 0.74
Adventurous Eating [59,60,61]	2	1 to 4	4-point agreement rating ^B^	0.72	3.16 ± 0.68
Maternal Self-Perceptions					
Personal Organization [62]	4	1 to 5	5-point agreement rating ^L^	0.69	3.68 ± 0.82
Need for Cognition [63,64]	1	1 to 5	5-point agreement rating ^L^	*	3.49 ± 0.98
Parenting Self-Efficacy [65,66]	1	1 to 5	5-point agreement rating ^L^	*	4.1 ± 0.81
Stress Management [67]	2	1 to 4	4-point agreement rating ^D^	0.84	3.94 ± 0.76
Stress Management Self-Efficacy	2	1 to 4	4-point agreement rating ^D^	0.79	2.63 ± 1.01
Child Weight Cognitions		1 to 5			
Belief that Chubby Kids are Healthy	2	1 to 5	5-point agreement rating ^L^	0.65	2.70 ± 0.74
Concern for Child’s Overweight Risk [68]	3	1 to 5	5-point agreement rating ^L^	0.91	1.91 ± 1.03
Health Behavior Values [31,69,70,71,72]					
Importance of Physical Activity for Self	3	1 to 5	5-point agreement rating ^L^	0.82	3.49 ± 0.97
Importance of Physical Activity for Child	3	1 to 5	5-point agreement rating ^L^	0.68	3.83 ± 0.87
Encourages/Facilitates Child Physical Activity	5	1 to 5	5-point agreement rating ^L^	0.88	4.23 ± 0.66
Importance of Modeling Physical Activity to Child	2	1 to 5	5-point agreement rating ^L^	0.79	4.13 ± 0.82
Engages in Physical Activity with Child Frequently	2	0 to 7	8-point frequency scale ^M^	*	3.67 ± 1.85
Models Physical Activity to Child Frequently	2	0 to 7	8-point frequency scale ^M^	*	3.08 ± 1.22
Models Sedentary Behaviors Infrequently	2	0 to 7	8-point frequency scale ^M^	*	2.79 ± 2.18
Models Healthy Eating to Child	4	1 to 5	5-point agreement rating ^L^	0.56	3.51 ± 0.73
Belief that TV Positively Affects Child Learning	2	1 to 5	5-point agreement rating ^L^	0.85	3.89 ± 0.76
Talks Often with Child about TV	2	1 to 5	5-point agreement rating ^L^	0.50	3.24 ± 0.97
Limits Child Exposure to TV Commercials and Inappropriate Programs Shows	2	1 to 5	5-point agreement rating ^L^	0.50	3.67 ± 0.93
Limits Child to Educational TV	1	1 to 5	5-point agreement rating ^L^	*	3.52 ± 1.09
Home Interpersonal Characteristics					
Child Feeding Practices [68,69,70,71,73,74,75]					
Restricts Child Food Intake	2	1 to 5	5-point agreement rating ^L^	0.63	3.84 ± 0.86
Pressures Child to Eat	3	1 to 5	5-point agreement rating ^L^	0.69	2.17 ± 0.96
Maternal Control Over Child Food Access and Choices	7	1 to 5	5-point agreement rating ^L^	0.65	3.33 ± 0.52
Non-Acceptance of Food Waste	2	1 to 5	5-point agreement rating ^L^	0.61	3.05 ± 0.97
Instrumental Feeding Practices (uses food to reward children for eating a healthy food)	3	1 to 5	5-point agreement rating ^L^	0.73	2.63 ± 0.91
Non-Food Rewards (uses non-food (e.g., extra playtime) to reward children for eating a healthy food)	2	1 to 5	5-point agreement rating ^L^	0.65	2.90 ± 0.95
Allows Child to Independently Access Nutrient Dense Foods	5	0 to 5	yes/no	*	1.82 ± 1.74
Allows Child to Independently Access Low Nutrient Density Foods	6	0 to 6	yes/no	*	0.68 ± 1.32
Nutrient Dense Foods Stored in Locations Visible to Child	5	0 to 5	yes/no	*	2.44 ± 1.71
Low Nutrient Dense Foods Stored in Locations Visible to Child	6	0 to 6	yes/no	*	0.82 ± 1.35
Family Meal Patterns [70,76,77,78,79,80]					
Family Meal Frequency	3	0 to 21	0–7 days for breakfast, lunch, dinner; score is sum of 3 meals	*	13.64 ± 5.05
Importance of Family Meals	3	1 to 5	5-point agreement rating ^L^	0.70	4.52 ± 0.64
Positive Family Meal Atmosphere	3	1 to 5	5-point agreement rating ^L^	0.70	4.12 ± 0.85
Fast Food Eaten at Family Meals	1	0 to 7	days/week	*	0.93 ± 1.18
TV on During Family Meals	1	0 to 7	days/week	*	2.24 ± 2.48
Family Meals Eaten at Kitchen or Dining Table	1	0 to 7	days/week	*	4.69 ± 2.51
Family Meals Eaten in the Car	1	0 to 7	days/week	*	0.43 ± 1.16
Family Meal Planning	2	1 to 5	5-point agreement rating ^L^	0.70	3.40 ± 0.88
Time and Energy for Family Meals	2	1 to 5	5-point agreement rating ^L^	0.78	4.34 ± 0.85
Family Functioning and Maternal Engagement					
Family Support for Healthy Behaviors [81,82,83]	4	1 to 5	5-point agreement rating ^L^	0.81	4.40 ± 0.73
Family Conflict and Lack of Cohesion [84,85,86]	5	1 to 5	5-point agreement rating ^L^	0.84	1.83 ± 0.70
Household Disorganization [87,88]	3	1 to 5	5-point agreement rating ^L^	0.76	2.47 ± 0.92
Verbal Engagement with Children	1	1 to 5	5-point agreement rating ^L^	*	4.17 ± 0.93
Physical Engagement with Children	1	1 to 5	5-point agreement rating ^L^	*	4.74 ± 0.51
Home Physical Environment Characteristics					
Physical Activity [69,70,71,72,82,89]					
Physical Activity Availability	12	1 to 5	5-point agreement rating ^L^	0.72	3.78 ± 0.67
Physical Activity Accessibility †	2	1 to 5	5-point agreement rating ^L^	0.90	4.21 ± 1.14
Media Devices in the Home	6	0 to more than 10	Total devices ^N^	*	11.57 ± 4.21
Media Devices in Child’s Bedroom	7	0 to 7	Total # of media device types ^O^	*	1.39 ± 1.62
Daily Screentime Child Allowed	1	0 to 1440	minutes/day	*	495.14 ± 714.22
Food Availability					
Household Fruit and Vegetable Availability (serving/person/day) [52,90]	10	0 to 19.94	9-point servings scale ^P^	*	6.41 ± 2.53
Household Fatty/Salty Snack Availability (serving/person/day) [52,90]	4	0 to 32	9-point servings scale ^P^	*	8.37 ± 7.22
Household Sugar-Sweetened Beverage Availability (serving/person/day) [55,56]	4	0 to 4.57	9-point servings scale ^P^	*	1.87 ± 1.79
Household Breakfast Cereal Availability (serving/person/day) [52,90]	1	0 to 8	9-point servings scale ^P^	*	5.35 ± 2.72

Note: n/a = not applicable. * Cronbach’s alpha is not appropriate for the scale type or because the scale has <2 items. † *n* = 524. ^A^ Family affluence assessed by three questions asking the total number of cars, vans, or trucks the family owns (0 = none, 1 = 1 vehicle, 2 = 2 or more vehicles), # of times family traveled on vacation in past year (0 = never, 1 = 1 time, 2 = 2 times, 3 = 3 or more times), and whether participant had their own bedroom (1 = yes, 0 = no); items are summed. ^B^ 4-point Agreement Rating: definitely false, mostly false, mostly true, definitely true; scored 1 to 4, respectively. Items averaged with higher scores indicating greater expression of the trait. ^C^ 5-point Excellence Rating: poor, fair, good, very good, excellent; scored 1 to 5 respectively; higher score indicates better health. ^D^ 4-point Occurrence Rating: not at all, several days, more than half the days, nearly every day; scored 1 to 4, respectively; scale scores equals average of items with higher scores indicating greater expression of the behavior. ^E^ 5-point Frequency Rating: never, rarely, sometimes, often, very often; scored 1 to 5 respectively; scale score equals average of item scores with higher scores indicating greater weight teasing history. ^F^ 4-point Frequency Rating: not at all, slightly, moderately, a lot; scored 1 to 4 respectively; higher scores indicate greater body dissatisfaction. ^G^ 8-point Exercise Days/week: 0, 1, 2, 3, 4, 5, 6, and 7; days/week weighted by exercise intensity (weights of 1, 2, 3 for walking, moderate, and vigorous activity, respectively) and summed to create scale score; higher scale score indicates greater activity level. ^H^ 5-point rating scale: very good, good, okay, bad, and very bad; scored 1 to 5 respectively with higher scores indicate poorer sleep quality. ^I^ 6-point Fruit/Vegetable Servings Rating: <1 serving/week, 1 serving /week, 2 to 3 servings/week, 4 to 6 servings/week, 1 serving/day, 2 or more servings/day; scored 0 to 5 respectively; scale scoring algorithm is protected by copyright and described in detail elsewhere. Possible score range = 0 to 12.17. ^J^ 5-point Fatty Food Servings Rating: 1 time/month or less, 2 to 3 times/month, 1 to 2 times/week, 3 to 4 times/week, 5 or more times/week; scored 0 to 4 respectively; scale scoring algorithm is protected by copyright and described in detail elsewhere. ^K^ 9-point Beverage Servings Rating: <1 time/week, 1 day/week, 2 days/week, 3 days/week, 4 days/week, 5 days/week, 6 days/week, 7 days/week, >1 time/day; scored 0 to 8 respectively. Possible score ranges for Sugar-Sweetened Beverages = 0 to 4.6; Milk = 0 to 8. ^L^ 5-point Agreement Rating: strongly disagree, disagree, neither agree nor disagree, agree, strongly agree; scored 1 to 5 respectively; scale score equals average of item scores with higher scale score indicating greater expression of the trait. ^M^ 8-point Modeling Days/week: 0 (almost never), 1, 2, 3, 4, 5, 6, and 7; days averaged to create scale score with higher score indicating more frequent modeling. ^N^ 11-point Media Device Count: 1 = 1 to 10 = 10, 11 = more than 10; scale score equals sum of items; higher score indicates greater number of media devices. Possible score range = 0 to 66. ^O^ Sum of 7 media devices found in child’s bedroom (i.e., TV, DVD player, Computer/Laptop, Smarphone/Tablet/LeapPad, video game devices (Nintendo DS, XBoxKinect), and Internet access). ^P^ 9-point Household Servings Rating: <1 time/week, 1 day/week, 2 days/week, 3 days/week, 4 days/week, 5 days/week, 6 days/week, 7 days/week, >1 time/day; scored 0 to 8 respectively. Possible score ranges for fruits/vegetables (0 to 19.94), salty/fatty snacks (0 to 32), sugar-sweetened beverages (0 to 4.57), and breakfast cereal (0 to 8) servings/household/member/day.

**Table 2:** Selection Rationale for Single Variable from Multicollinear Variable Groups.

Multicollinear Variable Group	Variable Most Highly and Significantly Correlated with BMI Was Retained in Further Analysis
Depression Severity, Health-related Quality of Life, and Stress Management	Health-Related Quality of Life
Disinhibited Eating and Emotional Eating	Emotional Eating
Importance of Physical Activity for Self with (1) Maternal Physical Activity Level and (2) Importance of Modeling Physical Activity to Child	Importance of Physical Activity for Self
Encourages/Facilitates Child Physical Activity with (1) Importance of Physical Activity for Child and (2) Importance of Modeling Physical Activity to Child	Encourages/Facilitate Child Physical Activity (Note: Importance of Modeling Physical Activity to Child had a higher correlation with BMI; however, it could not be selected because it is intercorrelated with Importance of Physical Activity for Self; see above)
Models Physical Activity to Child Frequently with (1) Maternal Physical Activity Level and (2) Mother: Child Co-Physical Activity	Models Physical Activity to Child Frequently
Instrumental Feeding Practices and Non-Food Rewards	Instrumental Feeding Practices
Allows Child to Independently Access Nutrient Dense Foods and Nutrient Dense Foods Stored in Locations Visible to Child	Neither variable was significantly correlated with BMI (none included)
Allows Child to Independently Access Low Nutrient Density Foods and Low Nutrient Dense Foods Stored in Locations Visible to Child	Low Nutrient Dense Foods Stored in Locations Visible to Child
Family Meals Eaten at Kitchen or Dining Table and TV on During Family Meals	Family Meals Eaten at Kitchen or Dining Table
Positive Family Meal Atmosphere and Household Disorganization	Neither variable was significantly correlated with BMI (none included)
Importance of Family Meals and Time and Energy for Family Meals	Importance of Family Meals
Household Fruit and Vegetable Availability with (1) Fruit and Vegetable Intake and (2) Household Sugar-sweetened Beverage Availability	Household Fruit and Vegetable Availability

**Table 3:** Binomial Logistic Regression of Variables Associated with Maternal Obesity (*n* = 550).

Dependent Variable: Maternal Obesity ^†^					
Independent Variables	B ^‡^	SE ^#^	Odds Ratio	95% CI	*p*-value
Race (black or African American)	1.25	0.41	3.48	(1.56, 7.70)	0.002
Education Level (some college or less)	0.61	0.26	1.83	(1.09, 3.07)	0.021
Number of Children in Household	0.32	0.12	1.38	(1.08, 1.75)	0.010
General Health Rating ^a^	0.89	0.17	2.43	(1.73, 3.41)	<0.001
Perception of Weight Teasing History	0.52	0.11	1.69	(1.35, 2.11)	<0.001
Body Dissatisfaction	0.91	0.14	2.29	(1.91, 3.25)	<0.001
Primary Relative with History of Obesity	0.71	0.25	2.04	(1.25, 3.23)	0.004
Concern for Child’s Overweight Risk	0.28	0.13	1.32	(1.03, 1.69)	0.026
R					
Cox and Snell R Square	0.374				
Nagelkerke R Square	0.531				
**Tests of Model Coefficients**	**DF** *	**χ^2^**	***p*-value**		
	8	257.92	<0.001		

^‡^ Beta coefficient. * DF = Degrees of Freedom. ^#^ Standard Error. ^†^ Maternal obesity defined at body mass index ≥ 30. ^a^ Higher scores indicate poorer perceptions of health status.

**Table 4:** ANOVA of Sociodemographic, Intrapersonal, Interpersonal and Home Environment Characteristics among Obesity Risk Score Tertiles of Non-Obese Participants.

	Obesity Risk Score Tertiles of Non-Obese Participants (*N* = 386)				
	Low Risk (*n* = 120)	Moderate Risk (*n* = 135)	High Risk (*n* = 131)		
Weighted Score Cut-offs	0 to 1.12	1.13 to 2.07	≥2.08	ANOVA *	
	Mean ± SD or *N* (%)	Mean ± SD or N (%)	Mean ± SD or *N* (%)	*F* or χ^2^	*p*-value
Obesity risk score (possible score range 0–5.49)	0.58 ± 0.36 †^,a^	1.52 ± 0.27 ^b^	2.78 ± 0.53 ^c^	953.53	<0.001
Sociodemographic Characteristics					
Maternal Employment				5.03	0.284
Do not work	56 (46.7)	73 (54.1)	78 (59.5)		
Part-time (less than 30 h/week)	24 (20.0)	28 (20.7)	21 (16.0)		
Full-time (30 or more h/week)	40 (33.3)	34 (25.2)	32 (24.4)		
Number of Children in Household	2.07 ± 0.97	2.21 ± 1.02	2.22 ± 0.96	0.94	0.392
Family Affluence Score	6.09 ± 1.49 ^a^	5.63 ± 1.61 ^b^	5.49 ± 1.53 ^b^	5.16	0.006
Food Insecurity Risk	1.38 ± 1.76 ^a^	1.78 ± 1.80	2.27 ± 1.82 ^b^	7.81	<0.001
Weight Status					
Mother’s BMI	22.15 ± 2.64 ^a^	22.93 ± 3.03 ^a^	25.19 ± 3.05 ^b^	37.36	<0.001
Child’s BMI percentile (*n* = 339)	59.85 ± 34.58	62.07 ± 34.76	65.45 ± 35.45	0.89	0.413
Health-Related Assessments					
Health-Related Quality of Life (# of unhealthy days)	0.94 ± 1.57 ^a^	1.61 ± 2.51 ^a^	4.16 ± 5.49 ^b^	27.97	<0.001
Depression Severity	0.57 ± 1.02 ^a^	0.85 ± 1.41 ^a^	1.33 ± 1.48 ^b^	10.68	<0.001
Age at Birth of First Child (years)	25.92 ± 4.72 ^a^	24.30 ± 5.12 ^a^	23.92 ± 5.59 ^b^	5.08	0.007
Primary Relative with History of Diabetes (% yes)	23 (19.2)	21 (15.6)	38 (29.0)	7.64	0.022
Intrapersonal Characteristics					
Maternal Weight-Related Behaviors					
Physical Activity Level	17.43 ± 9.78	15.93 ± 9.49	14.65 ± 10.50	2.46	0.087
Screentime (minutes/day)	216.25 ± 214.87	270.78 ± 249.99	277.67 ± 251.36	2.43	0.089
Sleep Duration (hours/day)	7.49 ± 1.97	7.22 ± 1.85	6.87 ± 1.89	3.31	0.038
Sleep Quality	3.58 ± 0.87 ^a^	3.46 ± 0.82 ^a^	3.03 ± 0.83 ^b^	15.00	<0.001
Fruit and Vegetable (servings/day)	4.98 ± 2.37	4.84 ± 2.30	4.24 ± 2.29	3.65	0.027
% Calories from Total Fat	36.83 ± 5.36	37.61 ± 6.55	36.98 ± 5.87	0.64	0.530
Milk (servings/day)	4.18 ± 3.19	4.31 ± 2.95	3.33 ± 3.11	3.79	0.023
Sugar-Sweetened Beverage (servings/day)	0.70 ± 0.78	0.95 ± 1.06	0.93 ± 0.86	2.83	0.060
Maternal Eating Styles					
Disinhibited Eating	1.77 ± 0.77	1.95 ± 0.76	2.00 ± 0.73	2.25	0.040
Emotional Eating	1.73 ± 0.74 ^a^	1.87 ± 0.80 ^a^	2.19 ± 0.88 ^b^	10.95	<0.001
Dietary Restraint Eating	2.31 ± 0.79	2.47 ± 0.79	2.49 ± 0.69	2.27	0.105
Adventurous Eating	3.21 ± 0.69	3.16 ± 0.66	3.12 ± 0.67	0.52	0.597
Maternal Self-Perceptions					
Personal Organization (self-effectiveness)	3.91 ± 0.75 ^a^	3.79 ± 0.79 ^a^	3.49 ± 0.83 ^b^	9.31	<0.001
Need for Cognition	3.66 ± 1.01	3.47 ± 0.94	3.37 ± 0.98	2.76	0.065
Parenting Self-Efficacy	4.23 ± 0.71 ^a^	4.23 ± 0.75 ^a^	3.95 ± 0.82 ^b^	5.71	0.004
Stress Management	4.19 ± 0.54 ^a^	4.02 ± 0.71 ^a^	3.79 ± 0.83 ^b^	10.41	<0.001
Stress Management Self-Efficacy	2.88 ± 1.01	2.51 ± 1.05	2.64 ± 0.99	1.43	0.242
Child Weight Cognitions					
Belief that Chubby Kids are Healthy	2.72 ± 0.75	2.79 ± 0.68	2.61 ± 0.72	1.95	0.143
Health Behavior Values					
Importance of Physical Activity for Self	3.90 ± 0.92 ^a^	3.75 ± 0.81 ^a^	3.34 ± 0.92 ^b^	13.94	<0.001
Importance of Physical Activity for Child	4.03 ± 0.78 ^a^	3.85 ± 0.85	3.69 ± 0.87 ^b^	5.12	0.006
Encourages/Facilitates Child Physical Activity	4.37 ± 0.67	4.29 ± 0.61	4.16 ± 0.64	3.44	0.033
Importance of Modeling Physical Activity to Child	4.35 ± 0.82 ^a^	4.26 ± 0.71	4.07 ± 0.76 ^b^	4.61	0.010
Engages in Physical Activity with Child Frequently	3.92 ± 1.90	3.61 ± 1.91	3.44 ± 1.80	2.11	0.123
Models Physical Activity to Child Frequently	3.55 ± 1.10 ^a^	3.13 ± 1.21 ^b^	2.93 ± 1.18 ^b^	9.14	<0.001
Models Sedentary Behaviors Infrequently	3.54 ± 2.11 ^a^	2.84 ± 2.08 ^b^	2.75 ± 2.27 ^b^	5.00	0.007
Models Healthy Eating to Child	3.72 ± 0.75 ^a^	3.62 ± 0.63	3.43 ± 0.69 ^b^	5.74	0.003
Belief that TV Positively Affects Child Learning	3.91 ± 0.68	3.91 ± 0.8	3.78 ± 0.78	1.30	0.274
Talks Often with Child about TV	3.38 ± 1.01	3.27 ± 0.94	3.26 ± 0.94	0.60	0.548
Limits Child Exposure to TV Commercials and Inappropriate Programs Shows	3.77 ± 0.90	3.71 ± 0.86	3.66 ± 0.93	0.48	0.621
Limits Child to Educational TV	3.68 ± 1.03 ^a^	3.64 ± 1.02 ^a^	3.31 ± 1.09 ^b^	4.66	0.010
Home Interpersonal Characteristics					
Child Feeding Practices					
Restricts Child Food Intake	3.85 ± 0.88	3.91 ± 0.84	3.83 ± 0.83	0.31	0.737
Pressures Child to Eat	2.22 ± 1.04	2.18 ± 0.95	2.15 ± 0.89	0.18	0.837
Maternal Control Over Child Food Access and Choices	3.44 ± 0.49	3.38 ± 0.55	3.30 ± 0.48	2.40	0.092
Non-Acceptance of Food Waste	3.12 ± 0.96	3.26 ± 0.94	3.05 ± 0.89	1.75	0.175
Instrumental Feeding Practices (uses food to reward children for eating a healthy food)	2.74 ± 0.97	2.77 ± 0.93	2.55 ± 0.87	2.01	0.136
Non-Food Rewards (uses non-food [e.g., extra playtime] to reward children for eating a healthy food)	2.93 ± 1.00	2.95 ± 0.98	2.89 ± 0.86	0.16	0.854
Allows Child to Independently Access Nutrient Dense Foods	1.74 ± 1.74	1.96 ± 1.68	1.73 ± 1.78	0.75	0.475
Allows Child to Independently Access Low Nutrient Density Foods	0.48 ± 1.04	0.78 ± 1.43	0.70 ± 1.40	1.81	0.166
Nutrient Dense Foods Stored in Locations Visible to Child	2.30 ± 1.72	2.42 ± 1.71	2.61 ± 1.78	1.03	0.360
Low Nutrient Dense Foods Stored in Locations Visible to Child	0.58 ± 1.21	0.79 ± 1.39	0.94 ± 1.36	2.26	0.106
Family Meal Patterns					
Family Meal Frequency (per week)	14.25 ± 5.00	13.92 ± 4.49	12.97 ± 5.09	2.39	0.093
Importance of Family Meals	4.64 ± 0.57	4.53 ± 0.64	4.42 ± 0.65	3.69	0.026
Positive Family Meal Atmosphere	4.18 ± 0.86	4.09 ± 0.88	4.08 ± 0.79	0.57	0.569
Fast Food Eaten at Family Meals					
TV on During Family Meals (days/week)	1.53 ± 2.17	2.22 ± 2.53	2.24 ± 2.43	3.55	0.030
Family Meals Eaten at Kitchen or Dining Table (days/week)	5.38 ± 2.23 ^a^	4.82 ± 2.48	4.42 ± 2.42 ^b^	5.13	0.006
Family Meals Eaten in the Car	0.51 ± 1.29	0.54 ± 1.39	0.35 ± 0.90	0.92	0.401
Family Meal Planning	3.53 ± 0.93	3.46 ± 0.9	3.4 ± 0.80	0.70	0.496
Time and Energy for Family Meals	4.46 ± 0.78	4.33 ± 0.85	4.22 ± 0.92	2.57	0.078
Family Functioning and Maternal Engagement					
Family Support for Healthy Behaviors	4.54 ± 0.74	4.34 ± 0.86	4.37 ± 0.65	2.56	0.079
Family Conflict and Lack of Cohesion	1.65 ± 0.62 ^a^	1.76 ± 0.64	1.91 ± 0.66 ^b^	5.29	0.005
Household Disorganization	2.32 ± 0.96	2.48 ± 0.92	2.55 ± 0.87	2.03	0.133
Verbal Engagement with Children	4.33 ± 0.84	4.15 ± 0.91	4.05 ± 1.01	2.91	0.056
Physical Engagement with Children	4.81 ± 0.44	4.67 ± 0.6	4.73 ± 0.51	2.11	0.122
Home Physical Environment Characteristics					
Physical Activity					
Physical Activity Availability	3.90 ± 0.68	3.82 ± 0.62	3.78 ± 0.64	1.12	0.327
Physical Activity Accessibility	4.42 ± 0.98	4.39 ± 1.16	4.12 ± 1.15	2.84	0.060
Media Devices in the Home (total number)	11.53 ± 4.29	11.09 ± 4.19	11.5 ± 3.71	0.49	0.613
Media Devices in Child’s Bedroom (total number)	1.05 ± 1.55	1.37 ± 1.70	1.63 ± 1.75	3.71	0.025
Daily Screentime Child Allowed (minutes/day)	342.00 ± 413.14	433.78 ± 642.76	509.54 ± 710.46	2.39	0.093
Food Availability					
Household Fruit and Vegetable Availability (serving/person/day)	6.96 ± 2.41 ^a^	6.80 ± 2.62	6.09 ± 2.44 ^b^	4.40	0.013
Household Fatty/Salty Snack Availability (serving/person/day)	9.21 ± 7.81	8.67 ± 7.73	8.07 ± 6.81	0.73	0.481
Household Sugar-Sweetened Beverage Availability (serving/person/day)	1.70 ± 2.02	2.21 ± 2.02	1.72 ± 1.51	3.15	0.044
Household Breakfast Cereal Availability (servings/person/day)	5.67 ± 2.78	5.39 ± 2.70	5.49 ± 2.52	0.36	0.698

* Analysis of Variance (ANOVA) for continuous variables and Chi-square analysis for categorical variables examined characteristic differences among the Obesity Risk Score Tertile groups (low risk, moderate risk, and high risk). Tukey post-hoc tests were conducted for characteristics that were statistically significant (*p* < 0.01) in the ANOVA to determine significant between group differences. † Differing superscript lowercase letters in the same row indicate significant (*p* < 0.05) between group differences as determined by Tukey post-hoc tests.
